# Editorial: Unlocking the microbial code: potential role in sarcoidosis pathogenesis and treatment

**DOI:** 10.3389/fimmu.2026.1796347

**Published:** 2026-02-19

**Authors:** Claudio Tana, Nicol Bernardinello, Paolo Spagnolo

**Affiliations:** 1 Internal Medicine Unit, Eastern Hospital of Manduria, Azienda Sanitaria Locale (ASL) Taranto, Manduria, Italy; 2 Respiratory Disease Unit, Department of Cardiac, Thoracic, Vascular Sciences and Public Health, University of Padova, Padova, Italy

**Keywords:** granulomatous inflammation, microbiota–immune interactions, multidisciplinary management, precision medicine, sarcoidosis

Sarcoidosis remains one of the most compelling unresolved clinical challenges. It is defined by non-caseating granulomatous inflammation and remarkable clinical heterogeneity (1, 2), but still lacks a unifying pathogenic framework (3). Despite substantial advances in immunology, purely immune-centered models have failed to fully explain why granulomatous inflammation emerges, persists, or resolves across patients and organs (3).

The Research Topic “Unlocking the Microbial Code: Potential Role in Sarcoidosis Pathogenesis and Treatment” was conceived to explore a converging line of evidence suggesting that sarcoidosis may arise from a dynamic and context-dependent interaction between the immune system and microbial ecosystems (4). The contributions gathered herein collectively point toward the microbiota—both intestinal and pulmonary—as an active immunological modulator capable of shaping macrophage behavior, T-cell polarization, and cytokine networks that underpin granuloma formation (5).

In this framework, the comprehensive review by Rizzi et al. critically integrates emerging metagenomic and immunological data, proposing that dysbiosis may represent a biologically meaningful component of disease susceptibility, phenotype, and progression rather than a secondary epiphenomenon.

From a translational perspective, the contribution by Ucciferri et al. further defines this paradigm by highlighting how antibiotic exposure can induce long-lasting perturbations of the host microbiota with potential downstream consequences for immune balance and inflammatory trajectories, raising timely and clinically relevant questions regarding antimicrobial stewardship in sarcoidosis.

At the tissue level, original research comparing lung granulomas in sarcoidosis and tuberculosis provides a crucial mechanistic bridge, revealing that sarcoid granulomas possess a distinct immune architecture and spatial organization despite histological similarities, underscoring that granulomatous inflammation is not a uniform endpoint but a finely tuned, context-specific immune structure. Complementary experimental evidence on pro-resolving mediators such as resolvin D2, although not disease-specific, reinforces the importance of resolution pathways in restoring immune homeostasis and suggests that failure to engage these mechanisms may contribute to persistent granulomatous inflammation.

Taken together, the studies in this Research Topic converge toward a more integrated and biologically nuanced model in which sarcoidosis might result, at least in a subset of patients, from the interplay between microbial signals, immune regulation, and tissue microenvironments (6). This conceptual evolution is closely aligned with recent advances emphasizing the need for a multidisciplinary approach to management and the integration of novel technologies in sarcoidosis care, where immunology, imaging, clinical phenotyping, and systems-based approaches converge to address several aspects of this complex disease (7).

**Figure 1:**
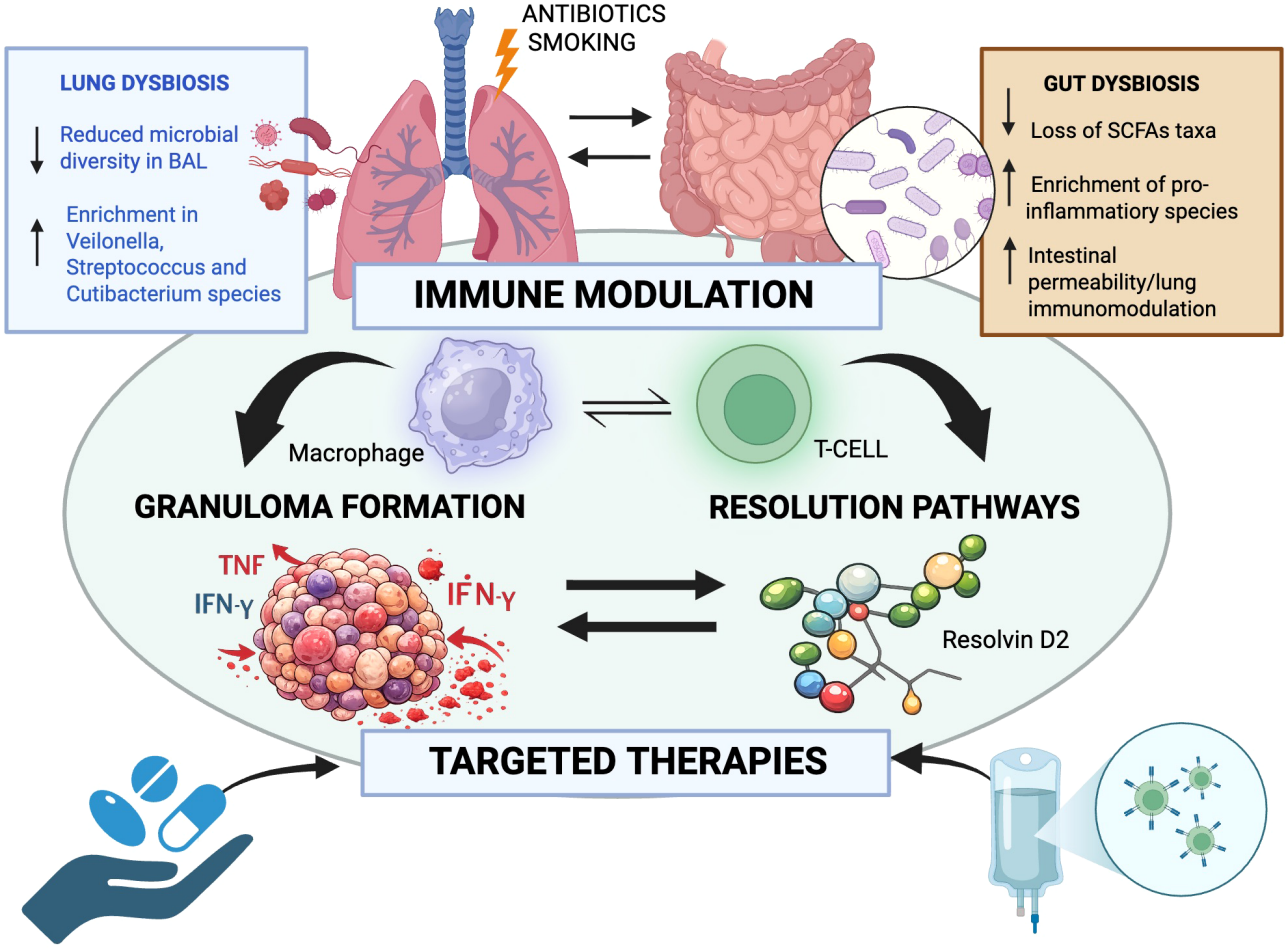
Provides a schematic overview of the pathogenetic hypotheses, and representative studies discussed in this special issue. Lung and gut dysbiosis along with the lung–gut axis might modulate immune responses, thus influencing the balance between granuloma formation and resolution. This integrated framework highlights how microbial alterations and immune modulation may inform targeted therapeutic strategies. (Figure created with Biorender. License number: IK29AAQCI8)..

In particular, emerging frameworks that incorporate advanced imaging modalities and refined therapeutic strategies—especially in high-risk organ involvement such as the heart, nervous system, and eye—underscore how deeper pathobiological insight can inform precision-oriented clinical decision-making (8–12). An overview of the key mechanisms, working hypotheses, and representative studies discussed by the articles included in this special issue is illustrated in **Figure 1**.

By bringing microbiome science into the core of sarcoidosis research and embedding it within a broader multidisciplinary and technology-driven perspective, this topic supports a shift from empiric, broad immunosuppression to targeted strategies aimed at restoring immune–microbial homeostasis and redefining how this complex and heterogeneous disease is understood and managed.
